# The implementation of nice guidance on venous thromboembolism risk assessment and prophylaxis: a before-after observational study to assess the impact on patient safety across four hospitals in England

**DOI:** 10.1186/1472-6963-13-203

**Published:** 2013-06-04

**Authors:** Alice G Bateman, Rod Sheaff, Susan Child, Olga Boiko, Obioha C Ukoumunne, Tim Nokes, Adrian Copplestone, Christian A Gericke

**Affiliations:** 1 PenCLAHRC, National Institute for Health Research, Plymouth University Peninsula Schools of Medicine and Dentistry, N6 ITTC Building, Tamar Science Park, Derriford, Plymouth PL6 8BX, UK; 2 PenCLAHRC, National Institute for Health Research, Exeter Medical School, Exeter, UK; 3 Plymouth Hospitals NHS Trust, Universities of Plymouth, Plymouth, UK; 4 Wesley Research Institute, University of Queensland School of Population Health, Queensland University of Technology School of Public Health, Brisbane, Australia

**Keywords:** Venous thromboembolism (VTE), Implementation strategies, NICE, Patient safety

## Abstract

**Background:**

Venous thromboembolism (VTE) is a major cause of morbidity and mortality in hospitalised patients. VTE prevention has been identified as a major health need internationally to improve patient safety. A National Institute for Health and Clinical Excellence (NICE) guideline was issued in February 2010. Its key priorities were to assess patients for risk of VTE on admission to hospital, assess patients for bleeding risk and evaluate the risks and benefits of prescribing VTE prophylaxis.

The aim of this study was to evaluate the implementation of NICE guidance and its impact on patient safety.

**Methods:**

A before-after observational design was used to investigate changes in VTE risk assessment documentation and inappropriate prescribing of prophylaxis between the year prior to (2009) and the year following (2010) the implementation of NICE guidance, using data from a 3-week period during each year. A total of 408 patients were sampled in each year across four hospitals in the NHS South region.

**Results:**

Implementation strategies such as audit, education and training were used. The percentage of patients for whom a VTE risk assessment was documented increased from 51.5% (210/408) in 2009 to 79.2% (323/408) in 2010; difference 27.7% (95% CI: 21.4% to 33.9%; p < 0.001). There was little evidence of change in the percentage who were prescribed prophylaxis amongst patients without a risk assessment (71.7% (142/198) in 2009 and 68.2% (58/85) in 2010; difference −3.5% (95% CI: -15.2% to 8.2%; p =0.56) nor the percentage who were prescribed low molecular weight heparin amongst patients with a contraindication (14% (4/28) in 2009 and 15% (6/41) in 2010; RD = 0.3% (95% CI: -16.5% to 17.2%; p =0.97).

**Conclusions:**

The documentation of risk assessment improved following the implementation of NICE guidance; it is questionable, however, whether this led to improved patient safety with respect to prescribing appropriate prophylaxis.

## Background

Venous thromboembolism (VTE) is a major, and often unrecognised cause of patient morbidity and mortality in hospitalised patients [1]. Pulmonary embolism (PE) accounts for 5-10% of hospital deaths and is, therefore, often quoted as the most preventable cause of death in hospital [1-5]. Hospitalised patients are at a 100 times greater risk than primary care patients [6] and between 25-30% of non-fatal VTEs occur in patients with prior hospitalisation [1].

The prevention of VTE has been identified as a major health need nationally and internationally to improve patient safety [5]. A recent multinational, observational, cross-sectional study carried out in 358 hospitals from 32 different countries (the ENDORSE study) showed that 51.8% of patients were at risk of VTE and only 50.2% of patients who were deemed to be at risk received prophylaxis [4]. A retrospective review of patients with a diagnosis of VTE was performed in 2010 in New Zealand and supported these findings. It demonstrated that 25% of patients with a VTE had been admitted to hospital in the preceding three months. Of these patients, two thirds had not received appropriate prophylaxis [7].

Several trials have been performed studying the use of single and multiple implementation strategies [5,8-10]. Single strategies such as passive dissemination are the least effective method of implementing guidelines, whereas combined systems of education, reminders, audit and feedback are believed more effective [5]. Many studies, however, presented short term data, so it is not known whether these implementation programmes had lasting effects [5,8]. In addition, many are examining the implementation of local guidelines.

This study presents data from the implementation of a new national guideline issued by the National Institute for Health and Clinical Excellence (NICE) in the UK in February 2010 (see below) [11].

NICE Clinical Guideline 92, 2010 [12]

Key priorities:

•Assess patients for risk of VTE on admission to hospital

•Assess patients for bleeding risk

•Weigh up benefits and risks of prescribing prophylaxis and prescribe if appropriate

Prophylactic measures to be taken if indicated:

•Pharmacological measures, e.g., fondaparinux sodium, LMWH, unfractionated heparin (UFH) in patients with renal failure

•Mechanical prophylaxis, e.g., anti-embolism stockings, foot impulse devices and intermittent pneumatic compression devices

General measures should also be taken:

•Early mobilisation

•Adequate hydration

### Objectives

The aim of this study was to evaluate the implementation of the NICE guideline across four hospitals in the NHS South of England region, and its impact on patient safety using the following outcome measures:

1. The percentage of patients for whom a risk assessment was documented.

2. The percentage of patients who received VTE prophylaxis amongst those who were not risk assessed.

3. The percentage of patients who received VTE prophylaxis amongst those with a contraindication to VTE prophylaxis.

The percentages were compared between the years prior to (2009) and following (2010) publication of the NICE guideline.

## Methods

### Study method

A before-after observational study design was used to evaluate the implementation of the NICE guideline on VTE prevention in four hospital sites. Two specialties were covered in this study; one with a relatively high proportion of elective patients (orthopaedics) and one with a high proportion of emergency admissions (general internal medicine).

### Sample selection

We sampled four hospitals to provide a maximum-variety sample of initially divergent models of VTE prevention activity because that would be likely to expose a range of approaches to implementing the NICE guideline, and a wide range of staff attitudes and motives. Our sampling frame was a regional census which reported current practice, the organisational context and VTE prevention activity for each hospital.

Each hospital approached the request to devise a sampling frame in different ways. Hospital A gave free access to admission data, however, Hospitals B, C and D would not release admission data but did provide a file sample of patient files for audit. Subsequent data analysis confirmed that in those hospitals where it was not possible to take our own sample, a random cross-section of files had been made available without ‘cherry-picking’ files to show 100% compliance with national VTE risk assessment guidelines.

The sample was systematically drawn from a list of all adult admissions of over 24 hours for a three-week period in October in the year prior to (2009) and the year following (2010) the NICE guideline publication. Data from 2009 were extracted as these pre-date protocol implementation, whilst 2010 data were used to cover the post-guidance implementation phase. October was chosen to minimise the impact of the four-monthly rotation of junior doctors and the seasonal inflow of tourists. Fifty one orthopaedic patient records and 51 general medicine patient records were sampled from each site in each of 2009 and 2010 (408 subjects overall in each year). This sample size is large enough to detect an increase from 50% in 2009 to 60% in 2010 in the proportion of patients for whom a risk assessment was documented, with 80% power at the 5% level of significance.

### Hospital characteristics

Table 1.

### Hospitals with Exemplar Status

Hospitals are given ‘Exemplar Status’ in the UK if they can show an existing track record of excellent VTE prevention and care. Hospitals are invited to join the ‘Exemplar Centre Network’ where they are able to share examples of good practice such as clinical best practice and educational and audit material, provide advice regarding VTE care, receive visitors and collaborate on clinical research into VTE. The network can also provide help and advice in relation to managing VTE prevention locally such as establishing hospital thrombosis committees [13].

Hospital A was awarded Exemplar Status in December 2009, recognising the extensive work it had completed towards good clinical care in the prevention of VTE. The Thrombosis Committee within this hospital was established in March 2006. The Trust demonstrated their commitment to VTE prevention in 2010, by putting in place a VTE prevention team, including a full time VTE Clinical Nurse Specialist, some designated senior doctor clinical time and audit/pharmacy time.

**Table 1:** Hospital characteristics

**Hospital site**	**A**	**B**	**C**	**D**
Population	500,000	450,000	350,000	200,000
Staff	6,000	5,200	6,000	3,900
Beds	940	750	850	550
Teaching Hospital	Yes	Yes	Yes	No
Foundation status	No	No	Yes	Yes
Exemplar Status prior to NICE guidance	Yes	No	No	Yes

Hospital D was also awarded Exemplar status in December 2009. It developed a simple means of documenting VTE risk with a ‘risk box’ on the front of the drug chart together with a recommendation for prophylaxis and space to document any contraindications to prophylaxis or reassessment of risk in early 2008. Risk decisions and prophylaxis were facilitated by a simple algorithm, which ensured that a high proportion of patients admitted to the Trust, would receive appropriate individualised measures to minimise their risk of VTE. Any VTE event was reviewed by the thrombosis committee and information fed back to the relevant clinical teams so that they had a better understanding of their own performance based on clinical outcomes.

### Data collection

Data on risk assessment, diagnosis and method of prophylaxis were extracted by a data collection instrument; designed mainly for ‘tick-box’ entry of standard data entry fields. Two experienced researchers reviewed the medical records, both of whom received training on reading medical records from a qualified practitioner before starting the data extraction. Qualitative data were collected from key informants at various levels of the medical and management hierarchies. Forty-four semi-structured interviews were completed with 50 interviewees across the four sites over an 18 month period during 2010 and 2011. The interviews described the pre-NICE 2009 systems and reported how each hospital had responded to the 2010 NICE guidance and what factors appeared to facilitate or hinder compliance.

### Analysis

The Chi-squared test was used to compare the following binary outcomes between 2009 and 2010: (1) whether patients had a documented risk assessment, (2) whether patients who were not risk assessed received VTE prophylaxis regardless, and (3) whether patients with a contraindication to VTE prophylaxis received it. The percentages with these traits are reported for each year, both overall and within each site, and the 95% confidence interval for the overall change in percentage. Tests of interaction were used to quantify evidence of differential change across the four sites. Statistical analyses were carried out using Stata software.

For the qualitative assessments, a semi-structured interview schedule was used starting with junior medical staff and working up the medical and managerial hierarchies.

### Ethical approval

Ethical approval was not needed for this study as it was deemed a service evaluation by the local NHS Research and Development Service.

## Results

### Risk assessment and prophylaxis prescribing

There was strong evidence of an increase in the percentage of patients for whom a risk assessment for VTE prophylaxis was documented after implementation of the NICE guideline (absolute increase across all sites = 27.7%; 95% CI: 21.4% to 33.9%; p < 0.001) (Table 2). Risk assessment documentation increased in all four hospitals, although a test of interaction indicated that the amount of change differed among them (p < 0.001).

**Table 2:** Risk assessment, prophylaxis use and low-molecular weight heparin use by hospital in 2009 and 2010

**Hospital**	**Year**	**Risk assessment,%**	**Prophylaxis use,%**	**LMWH use,%**
All	2009	51.5	80.4	49.8
	2010	79.2	85.8	60.3
A	2009	43.1	91.2	67.6
	2010	90.2	97.1	60.8
B	2009	58.8	76.5	63.7
	2010	83.3	82.4	52.9
C	2009	32.4	71.6	24.5
	2010	67.6	79.4	67.6
D	2009	71.6	82.4	43.1
	2010	75.5	84.3	59.8

*sample size is 102 for each hospital and year.

Data in each year collected over a 3-week period.

LMWH: low-molecular weight heparin.

The percentage of patients receiving any form of prophylaxis is also shown in Table 2. Again, there was evidence at the 5% level of an improvement overall (absolute increase across all sites = 5.4%; 95% CI: 2.6% to 10.5%; p = 0.04), with each hospital showing an improvement. Although the use of low molecular weight heparin (LMWH) increased overall (absolute increase across all sites = 10.5%; 95% CI: 3.8% to 17.3%; p = 0.002), it decreased in hospitals A and B; a test of interaction indicated evidence that the amount of change differed across the hospitals (p < 0.001).

### Contraindications and documented risk assessment

The number of patients who were not risk assessed decreased. However, the percentage of patients who received prophylaxis without documentation of a risk assessment only decreased slightly from 71.7% (142/198) to 68.2% (58/85) and there was little evidence of a true change (absolute decrease = 3.5%; 95% CI: -8.2% to 15.2%; p = 0.56). The percentage of patients who received LMWH when a contraindication was present was unchanged (14% (4/28) in 2009 versus 15% (6/41) in 2010; RD = 0.3%; 95% CI: -16.5% to 17.2%; p =0.97).

**Figure 1:**
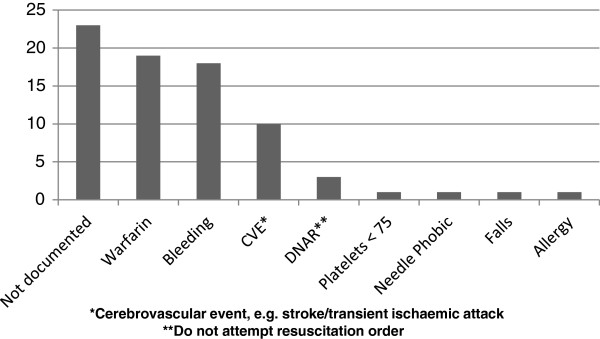
Documented reasons for contraindications to VTE prophylaxis.

We also examined the different contraindications documented. Sixty nine of the 816 patients (8.5%) were documented to have a contraindication to VTE prophylaxis in total. Of these, the three main reasons given were: 1) taking warfarin concomitantly (n = 19, 28%), 2) at risk of bleeding (n = 18, 26%), and 3) possible cerebrovascular event (n = 10, 14%). A few questionable reasons were also given, such as ‘needle phobic’ (n = 1, 1%) or ‘at risk of falls’ (n = 1, 1%) (see Figure 1). In 33% of cases (n = 23) no reason for contraindication was documented.

### Implementation strategies

The interviews with hospital professionals were transcribed and analysed using thematic content analysis. Findings demonstrated that the NICE guideline ensured a more systematic approach to VTE risk assessment and prescribing of prophylaxis in all four hospitals studied.

#### Policy and leadership

All hospitals included in this study had local policies prior to implementation of the NICE guideline and adapted their protocols to include the NICE recommendations, including clearly defined roles and responsibilities for staff. For example, Hospital A favoured universal prophylaxis with an ‘opt-out’ strategy, where all patients received prophylaxis unless contraindicated. Hospital B used multiple guidelines for VTE prevention, where each department had its own protocol. Hospital C emphasised early risk assessment with selective prescribing of prophylaxis, where only patients with a clear clinical need for prophylaxis received any. This probably explains the low percentage of prophylaxis prescribing in that hospital.

All hospitals created dedicated multi-disciplinary VTE committees with the consultants and clinical managers as champions:

‘*The VTE committee is quite robust and it has then sparked interest and support from the Trust Board, which has been really the key, and I think Dr N, a lead haematologist has been given an actual recognised role that’s also, you know, properly sort of remunerated, and securing a post for a VTE nurse has also been a key thing’* (Consultant, Hospital A).

Hospital D also included patients in the VTE committee in order to gain an additional perspective.

#### Risk assessment

The NICE guideline stipulated that all risk assessments were to be documented and all patients were to receive appropriate prophylaxis. NICE recommended that the tools were clear, easy to use and incorporated into another document to aid compliance. All sites incorporated the risk assessment into the drug charts used throughout the hospital. For example:

*‘In terms of the risk assessment that takes place when every patient is, or it should take place when every patient is admitted, because every drug chart in the hospital has a risk assessment box on it that inevitably has to be performed by the junior doctor on admission’* (Pharmacist, Hospital B).

#### Measurement and audit

All hospitals in our study conducted regular audits to measure compliance with risk assessment and presented results to the hospital staff at local meetings. Interviewees also commented on the importance of monitoring the link between risk assessments and prescribing appropriate prophylaxis:

*‘Our VTE risk assessment audit picks up on that because we look at how many patients have been prescribed Dalteparin and based on the fact that they have been categorised as being high risk, I would say we are running at about 97% prescribed Dalteparin. The patients who have not been prescribed Dalteparin normally have a documented reason why they have not received it’ (*Pharmacist, Hospital D).

Some hospitals also conducted ward-based audits, which ensured transparency of results throughout the specialities and hospitals promoting awareness and education about VTE.

#### Staff education and training

Hospitals A, C and D created a ‘link’ role, in the form of a VTE specialist nurse with a role in education and communication between medical, nursing and managerial staff. Hospital B is currently looking into employing a lead nurse.

Training in VTE risk and prophylaxis was included in the staff/trust induction of all hospitals. Hospital B made the completion of an online learning package regarding VTE risk mandatory within 48 hours of joining the trust. This trust also endorsed the use of an electronic whiteboard or ‘smartboard’, which obliged junior doctors to fill in the risk assessment for VTE, making it easier for consultants to see which patients had or had not been risk assessed for VTE prophylaxis. Hospital C developed a series of educational interventions and consultations across the Trust:

*‘There are a large number of training trackers, and each group will have mandatory ones to take; mandatory training trackers… There have been seminars, we moved from Tinzaparin to a single low molecular heparin in this trust and we have a road show and seminars for people and an education process around that, so those seminars have happened. We have our audit meetings where things get presented out and so we have audit days which includes talking about VTE’ (*Consultant, Hospital C).

Hospital D adopted a multimedia approach to educate and remind staff about VTE prophylaxis, e.g., screen savers, posters, printed leaflets in payslips etc.

## Discussion

### Main findings

All four hospitals showed a commitment to reduce avoidable VTEs and associated deaths. Our results show an improvement in the percentage of patients who are risk assessed for VTE across all hospitals. Hospital C, which showed the greatest increase, had no formal method of recording prophylaxis use and risk assessment before NICE guideline implementation. The hospital that showed the least improvement (hospital D), had exemplar status before the publication of the NICE guideline and already had high levels of risk assessment prior to it. The other exemplar site (hospital A) showed a dramatic improvement in risk assessment documentation with the introduction of the NICE guideline. This hospital (hospital A) previously had an ‘opt-out’ system; every patient received prophylaxis unless a contraindication was present. Clinicians did not formally record the risk assessment, hence relatively low levels were recorded in 2009. In 2010 it was the best site for recording risk assessment.

### Patient safety

We used the measures ‘the percentage of patients who received VTE prophylaxis amongst those who were not risk assessed’ and ‘the percentage of patients who received VTE prophylaxis amongst those with a contraindication to VTE prophylaxis’ to assess patient safety as prescribing in this group can potentially have catastrophic consequences for the patient. There was little evidence of a reduction in the percentage of patients prescribed prophylaxis who had not been risk assessed or for whom prophylaxis was contraindicated. The NICE guideline therefore did not appear to have an impact on the safety of these patients, although the large confidence interval widths indicate palpable uncertainty about the true change in inappropriate prophylaxis use.

#### Strengths of the study

We were able to use the dissemination of a national guideline to evaluate implementation strategies across four diverse hospital sites, which made a before-after observational study the strongest practicable study design for answering our research questions.

#### Limitations of the study

We chose the outcomes for this study to show if the guideline had improved patient safety. We acknowledge that there are other outcomes, which could also show this, such as ‘VTE attributable death’, but it was not possible to record this information.

Although before-after observational study was the best design, we recognise that it is not of as high methodological rigour as a randomised controlled trial and does not control for secular changes that may have occurred in the absence of guideline introduction.

Although recording bias is a possibility we would suggest that this is unlikely. The researchers used a standard data extraction sheet for all data recording and data were lifted from medical records and recorded word for word.

We were only able to present short term post-implementation data (i.e., 8 months after the implementation of the NICE guideline) and therefore do not know if these changes can be sustained or indeed improved in the long term.

The VTE risk of the patient populations may have been marginally different between 2009 and 2010. This should not have an impact on the percentage of patients that were risk assessed, however, as every patient, regardless of VTE, should have been risk assessed in line with the NICE guideline.

Finally, the study was conducted across only four hospitals and this is too small a sample from which to generalise the findings to the full spectrum of hospitals in England.

## Conclusions

National guidelines are effective at ensuring that hospitals promote evidence-based practice. The NICE guideline ‘*Venous thromboembolism - reducing the risk’* was implemented by all the hospitals in our study sample and more patients were risk assessed and prescribed prophylaxis following the introduction of the guideline. It is questionable, however, if this improved patient safety with regard to the ‘high risk’ patients (i.e. in those without a risk assessment or in those with a contraindication).

A multifaceted approach seems essential to continue the implementation and to ensure that all hospitals maintain and improve risk assessment rates. More importantly, there needs to be a shift of focus from risk assessment documentation to appropriate prescribing of prophylaxis which is the aim of the NICE guideline.

## Competing interests

All authors declared that they have no competing interests.

## Authors’ contributions

CG and RS conceived the study. AC and TN participated in design and the coordination of the study. SC and OB were responsible for data collection. OU performed the statistical analysis. AB drafted the manuscript. All authors read and approved the final manuscript.

## Pre-publication history

The pre-publication history for this paper can be accessed here:

http://www.biomedcentral.com/1472-6963/13/203/prepub
